# The Clinicopathological and Prognostic Value of PD-L1 Expression in Cholangiocarcinoma: A Meta-Analysis

**DOI:** 10.3389/fonc.2019.00897

**Published:** 2019-09-18

**Authors:** Gang Xu, Lejia Sun, Yunzhu Li, Feihu Xie, Xiaoxiang Zhou, Huayu Yang, Shunda Du, Haifeng Xu, Yilei Mao

**Affiliations:** ^1^Department of Liver Surgery, Peking Union Medical College Hospital, PUMC and Chinese Academy of Medical Sciences, Beijing, China; ^2^Department of Plastic Surgery, Peking Union Medical College Hospital, PUMC and Chinese Academy of Medical Sciences, Beijing, China

**Keywords:** biliary duct cancer, cholangiocarcinoma, programmed cell death ligand 1 (PD-L1), clinicopathology, prognosis, meta-analysis

## Abstract

**Background:** Recently, blockade of immune checkpoint has emerged as one of the most potential treatments for solid tumors. Programmed cell death ligand 1(PD-L1), a member of the B7 family of molecules, plays a crucial role in tumor immunobiology. However, the prognostic significance of PD-L1 in cholangiocarcinoma (CCA) patients remains controversial. This study aimed to inquire into the prognostic and clinicopathological significance of PD-L1 in CCA via a meta-analysis.

**Methods:** We searched PubMed, the Cochrane Library, Embase, Web of Science and Google Scholar up to April 2019, regardless of the region or language, for studies on the correlation between clinicopathology/prognosis and PD-L1 in patients with CCA. The pooled hazard ratios (HRs) and 95% confidence intervals (CIs) were calculated to investigate the prognostic significance of PD-L1 expression in cholangiocarcinoma. The odds ratios (ORs) were also determined to explore the association between PD-L1 expression and clinicopathological features.

**Results:** Our meta-analysis included 11 studies with 1,066 patients. The meta-analysis of these studies indicated a trend that high PD-L1 expression indicated a poor OS, but the result was not statistically significant (HR = 1.62, 95% CI [0.98–2.68], *p* = 0.063). For DFS, although the pooled result is not statistically significant, it trends toward being significant that high PD-L1 expression indicated improved DFS (HR = 0.80, 95% CI [0.62, 1.04], *p* = 0.092). In subgroup analyses, the results were not consistent across the subgroups that were divided based on the publication year (before 2018: HR = 1.92, 95% CI [1.34–2.75], *p* < 0.001; after 2018: HR = 1.42, 95% CI [0.70–2.89], *p* = 0.335). Moreover, PD-L1 expression in TCs significantly correlated with the AJCC TNM stage of CCA (OR = 0.52, 95% CI [0.27, 0.99], *p* = 0.09).

**Conclusion:** Our meta-analyses revealed that PD-L1 expressed in TCs was significantly correlated with the AJCC TNM stage of CCA. Based on the included studies, we found that PD-L1 indeed expressed in both TCs and ICs in CCA patients, raising the possibility of the use of anti-PD-1/PD-L1 therapy for CCA patients. In contrast, expression of PD-L1 did not seem to be associated with patient outcome in our study. The prognostic role of PD-L1 in CCA demands further investigation.

## Introduction

Cholangiocarcinoma (CCA) is one of the most common and aggressive malignant tumors that arises from bile duct epithelial cells. From the viewpoint of the anatomical location, CCAs consist of intrahepatic cholangiocarcinoma (iCCA) and extrahepatic cholangiocarcinoma (eCCA). eCCAs are subclassified as perihilar (pCCA) and distal (dCCA) tumors (1). Although surgical resection is considered to be the only curative treatment for CCA, many patients with CCA are diagnosed at an advanced stage and surgical resection is not an option for patients at advanced stages. However, the overall survival rate and therapeutic options for CCA have not improved in recent years (2–5). Therefore, novel adjuvant therapies for patients with CCA is in great demand.

In recent years, more and more studies have tried to investigate the interaction between the immune system and tumors. As a result, there have been remarkable advances in cancer immunotherapy research at present. In many malignant tumors, the immune cells (ICs) play a critical role in the tumor microenvironment (TME), with communication among natural killer (NK) cells, antigen presenting cells (APCs) such as dendritic cells (DC), and lymphocytes (T/B lymphocytes) allowing for e?ective tumor control (6). The immune response is a complex phenomenon based on the balance between activating and inhibitory pathways that regulate tumor-infiltrating lymphocytes (TILs) activity. Tumors can escape immunosurveillance by expressing immune checkpoints (7). The key role of the PD-L1/PD-1 axis, which is a major checkpoint pathway in tumor microenvironmental formation and immune escape, has been well established (8, 9). The programmed cell death ligand 1 (PD-L1), one of the ligands of programmed cell death receptor 1 (PD-1), plays an essential role in immune escape within the TME and in suppressing the generation and effector function of e?ector T cell in tumors (10). The monoclonal antibodies that block PD-1 or PD-L1 have emerged as one of the most potential treatments for solid tumors (11–13). As a member of the B7 family of molecules, PD-L1 expresses on the surface of tumor-associated antigen-presenting cells and malignant cells in numerous tumors that facilitates immune evasion via its interaction with PD-1 (14). The expression of PD-L1 correlates with poor prognosis in several human cancers (15–18). However, whether PD-L1 has prognostic value in patients with CCA remains controversial.

No systematic research has evaluated the predicted prognostic value of PD-L1 expression in CCA patients. For these reasons, a meta-analysis was performed to assess whether PD-L1 expression in tumor cells (TCs) and ICs were associated with the clinicopathological characteristics and prognosis in patients with CCA.

## Materials and Methods

### Literature Search and Selection Criteria

The PubMed, the Cochrane Library, Embase and Web of Science and Google Scholar was systematically searched up from 2,000 to April 2019, regardless of the region or language, for studies on the correlation between clinicopathology/prognosis and PD-L1 in patients with CCA. The following keywords were applied: (“PD-L1” or “CD274” or “B7-H1” or “programmed death ligand-1”) and (“biliary duct cancer” or “bile duct cancer” or “bile duct carcinoma” or “biliary duct carcinoma” or “cholangiocarcinoma”) and (“survival” or “outcome” or “prognosis” or “clinicopathology”). The reference lists of the retrieved papers were checked by us to ensure sensitivity of our search strategy. The “related articles” function was also used to broaden the search. The adopted inclusion criteria in the meta-analysis were as follows: (1) original articles; (2) reported the correlation of high or low PD-L1 level with clinicopathologic features, overall survival (OS) or disease-free survival (DFS); (3) studies provided sufficient information to estimate the hazard ratios (HR) for OS or DFS and a corresponding assessment of uncertainty (i.e., *p*-values, confidence interval (CI), variance or standard errors); (4) PD-L1 was detected in tumor tissues; and (5) full-text articles available in English. Studies inconsistent with the inclusion criteria were excluded. When duplicates were identified, only the latest or the single article provided with the most information was included.

### Data Extraction and Quality Assessment

Two reviewers independently (Xu G and Sun LJ) extracted the data, and any disputes between the two reviewers were settled by consensus involving a third reviewer (Li YZ). For each included study, the following data were extracted: publication year; the first author's name; country; ethnicity of the patients; number of the patients; tumor location; trial design; marker; therapy method; the type of tissue slide; PD-L1 site; PD-L1 assessment methods; PD-L1 antibody; cut-off definition; follow-up time; clinicopathologic parameters, such as gender, age, tumor size, tumor number, lymph node metastasis, perineural invasion, vascular invasion, resection margin, tumor differentiation, and AJCC TNM stage; OS or DFS; outcomes of univariate and/or multivariate analysis (including HRs, *p*-values and 95% CIs).

A quality assessment for all of the included studies was independently conducted by three investigators (Xu G, Li YZ, and Sun LJ). The Newcastle–Ottawa Quality Assessment Scale (NOS), which is a risk of bias assessment tool for observational studies that is recommended by the Cochrane Collaboration, was used to assess the quality of the included studies (19, 20). And any dispute was settled by discussion. The NOS is composed of the next three parameters of quality: selection, comparability, and outcome assessment. Each study was scored from 0–9 according to these parameters.

### Statistical Analysis

Positive or negative PD-L1 expression was defined according to the cut-off values provided by the authors. The effective value was evaluated based on the combination of HRs and their 95% CIs. We directly used crude values when HRs were reported in the original studies. If an explicit report of the survival and recurrence ratios was not accessible, the Kaplan–Meier curves were read by Engauge Digitizer version 4.1 to determine the survival information as described previously (21). Multivariate HRs and 95% CIs were chosen if both univariate and multivariate data were reported in an individual study to avoid confounding factors. The chi-squared test and *I*^2^ was applied to evaluate the statistical heterogeneity. A chi-squared *p* < 0.1 or an *I*^2^ statistic >50% was considered as statistically significant heterogeneity. If heterogeneity existed, we adopted a random-effects model to decrease the impact of heterogeneity on the results. If heterogeneity did not exist, a fixed-effects model was adopted instead. Subgroup analyses were conducted to investigate possible sources of heterogeneity. In addition, they helped to restrict studies to subgroups that might have inconsistent prognostic effects to determine the sensitivity of the conclusions. A cumulative analysis was conducted as well to investigate the trends in the results. Sensitivity analysis was performed to evaluate the stability of the results; a single study was deleted at every turn to observe the influence of the individual data on the overall results. The potential publication bias was evaluated by Egger's and Begg's tests. Meta-regression analysis was adopted to assess the effects of covariates on the pooled outcomes and heterogeneity among studies. The covariates included the publication year, sample size, proportion of males, ethnicity, positivity rate of PD-L1 and proportion of poorly differentiated tumors. *P* < 0.05 was regarded as significant. Odds ratios (ORs) with 95% CIs were used to assess the correlation of PD-L1 expression in TCs and ICs with clinicopathological features and TILs. All of the statistical analyses were performed using STATA version 14.0 (Stata Corporation; College Station, TX, USA). *p* < 0.05 were regarded to be statistically significant. All *p*-values and 95% CIs were two-sided.

## Results

### Study Selection

**Figure 1:**
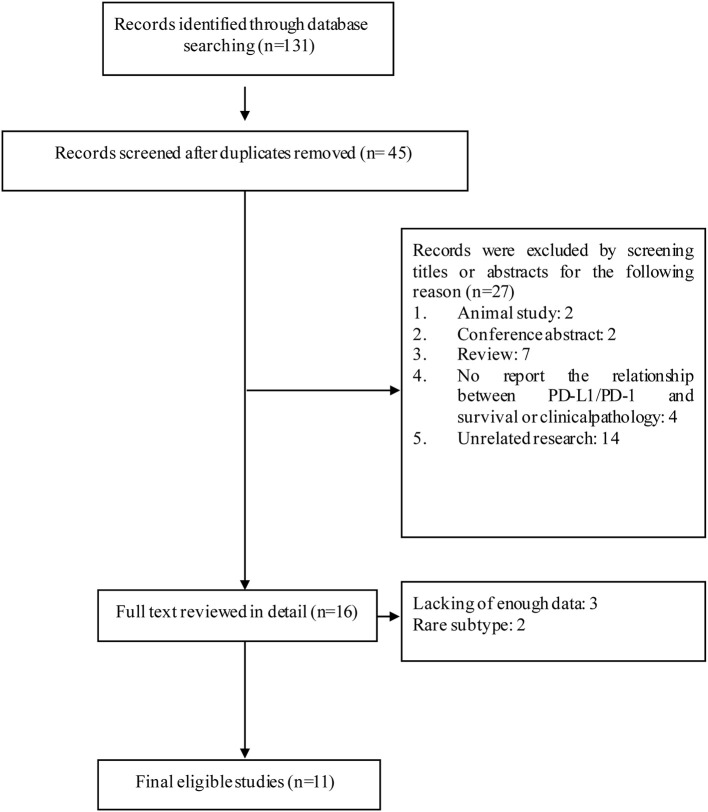
Flow diagram of the study selection process.

In the present study, 131 articles were identified with the initial searching strategy. Of these studies, 86 were removed because they were duplicate studies. A total of 27 records were excluded because they were deemed irrelevant based on the title and abstract. After thoroughly reviewing the full texts of the 40 potentially eligible articles, 13 trials meeting the inclusion criteria were included in the final analysis. A flowchart depicting the study selection strategy is shown in Figure 1.

### Characteristics of the Included Studies

The characteristics of the included studies are shown in Table 1. In total, 11 studies including 1,066 patients were enrolled in the pooled analysis. These studies were published between 2009 and 2019. All studies were retrospective cohort studies.

**Table 1:** The characteristics of studies included in the meta-analysis.

**Author**	**Country**	**Ethnicity**	**No**	**Tumor location**	**Marker**	**Trial design**	**Therapy**	**Method**	**Cut-off**	**Follow up (Months)**	**Outcome measured**
Gani et al. (22)	USA	Caucasian	54	Intra-CCA	PD-L1	RC	Surgery	IHC	5%	NR	OS
Ma et al. (23)	China	Asian	70	Ex-CCA	PD-L1	RC	Surgery	IHC	SID Score = 2	NR	OS
Fontugne et al. (24)	France	Caucasian	99	Mix-CCA	PD-L1	RC	Surgery	IHC	5%	NR	—
Sangkhamanon et al. (25)	Thailand	Asian	46	Mix-CCA	PD-L1	RC	Surgery	IHC	1%	NR	OS
Walter et al. (26)	Germany	Caucasian	69	Ex-CCA	PD-L1	RC	Surgery	IHC	H-score = 3	23 (Median)	OS
Kim et al. (27)	USA	Caucasian	34	Ex-CCA	PD-L1	RC	Surgery	IHC	5%	NR	OS, DFS
Zhu et al. (28)	China	Asian	192	Intra-CCA	PD-L1	RC	Surgery	IHC	5%	24 (Median)	OS, DFS
Ueno et al. (29)	Japan	Asian	117	Ex-CCA	PD-L1	RC	Surgery	IHC	H-score = 11	27 (Median)	OS
Kriegsmann et al. (30)	Germany	Caucasian	170	Mix-CCA	PD-L1	RC	Surgery	IHC	5%	NR	OS
Yu et al. (31)	China	Asian	62	Ex-CCA	PD-L1	RC	Surgery	IHC	TIS score = 3	NR	OS, DFS
Jing et al. (24)	China	Asian	153	Intra-CCA	PD-L1	RC	Surgery	IHC	5%	47.5 (Median)	OS

*CCA, Cholangiocarcinoma; IHC, Immunohistochemistry; RC, retrospective cohort; NR, Not reported; OS, overall survival; DFS, disease-free survival*.

Six studies (54.5%) were reported on Asian individuals, and 5 (45.5%) were reported on Caucasian individuals. Among the 11 studies, 10 studies reported a correlation between OS/DFS and PD-L1 expression, and 10 examined the connection between clinicopathologic features and PD-L1. The method for detecting PD-L1 was immunohistochemistry (IHC). The cut-off points of high-PD-L1 expression and the type of antibodies were heterogeneous. The study quality, as evaluated by the Newcastle-Ottawa quality assessment scale, ranged between five and seven. All enrolled studies were allocated scores >5 on NOS, suggesting that the methodology of studies possessed relatively high quality (Table 2).

**Table 2:** The Newcastle-Ottawa scale (NOS) quality assessment of the enrolled studies.

**References**	**Selection**				**Comparability**	**Outcome**			**Total**
	**Representativeness of the exposed cohort**	**Selection of the non-exposed cohort**	**Ascertainment of exposure**	**Demonstration that outcome of interest was not present at start of study**	Comparability of cohorts on the basis of the design or analysis (study adjusts for age^*^, ex^*^)	**Assessment of outcome**	**Was follow-up long enough for outcomes to occur**	**Adequacy of follow up of cohorts**	
Gani et al. (22)	-	^*^	^*^	-	^**^	^*^	^*^	^*^	7
Ma et al. (23)	-	^*^	^*^	-	^**^	^*^	^*^	^*^	7
Fontugne et al. (32)	-	^*^	^*^	-	^**^	^*^	-	-	5
Sangkhamanon et al. (25)	-	^*^	^*^	-	^*^	^*^	^*^	^*^	6
Walter et al. (26)	-	^*^	^*^	-	^**^	^*^	^*^	^*^	7
Kim et al. (27)	-	^*^	^*^	-	-	^*^	^*^	^*^	5
Zhu et al. (28)	-	^*^	^*^	-	^**^	^*^	^*^	^*^	7
Ueno et al. (29)	-	^*^	^*^	-	^**^	^*^	^*^	^*^	7
Kriegsmann et al. (30)	-	^*^	^*^	-	^*^	^*^	^*^	^*^	6
Yu et al. (31)	-	^*^	^*^	-	^*^	^*^	^*^	^*^	6
Jing et al. (24)	-	^*^	^*^	-	^**^	^*^	^*^	^*^	7

- , zero score;

* one score;

** *two scores*.

### Prognostic Effects of PD-L1 on Survival

#### PD-L1 in TCs OS and DFS

**Figure 2:**
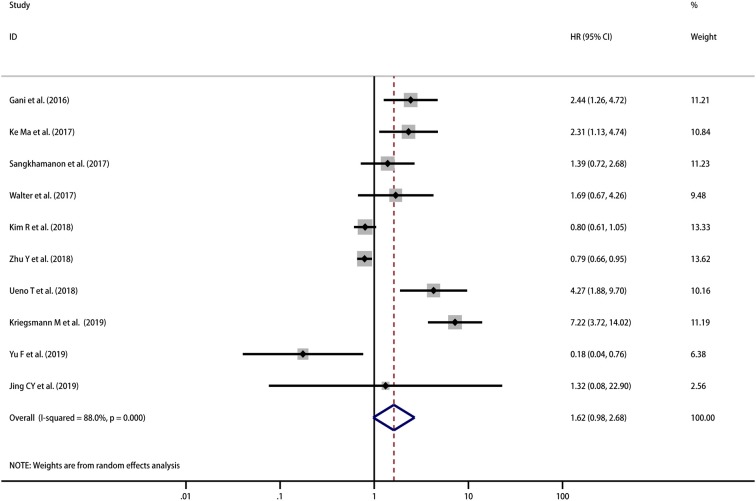
Meta-analysis of the correlation between PD-L1 expression in TCs and OS among patients with CCA.

A total of 967 patients from 10 studies were evaluated to examine the correlation between PD-L1 expression and OS. The meta-analysis of these studies showed a trend that high PD-L1 expression indicated a poor OS, but the result was not statistically significant (HR = 1.62, 95% CI [0.98–2.68], *p* = 0.063), and there was high heterogeneity among the studies (*I*^2^ = 88.0%, *p* < 0.001) (Figure 2). Therefore, we used a random effects model to estimate the pooled HRs and 95% CIs. As a result, according to various confounding factors, we carried out subgroup meta-analysis and meta-regression analysis to explore the possible sources of heterogeneity among the studies.

**Figure 3:**
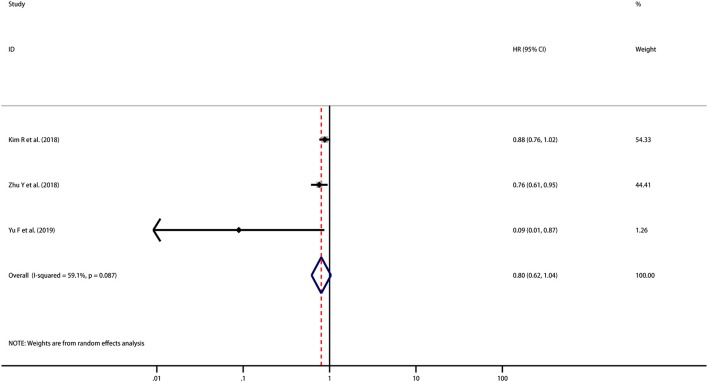
Meta-analysis of the correlation between PD-L1 expression in TCs and DFS among patients with CCA.

HRs for DFS were reported in 3 studies including 288 patients. Although the pooled result is not statistically significant, it trends toward being significant that high PD-L1 expression indicated improved DFS (HR = 0.80, 95% CI [0.62, 1.04], *p* = 0.092) with moderate heterogeneity (*I*^2^ = 59.1%, *p* = 0.087) (Figure 3). However, as the number of included studies was small, subgroup analyses and meta-regression analysis were not performed.

#### PD-L1 in ICs and OS

As the direct report of the survival of two studies was not available, the survival data of only two studies was determined by reading the Kaplan–Meier curves. Ma et al. reported that PD-L1 expression in ICs was associated with OS (HR = 2.47; 95% CI [1.23–4.96], *p* = 0.011) (23). However, in Walter's study, PD-L1 expression in ICs was not correlated with survival (HR = 0.86; 95% CI [0.43–1.70], *p* > 0.2) (26). Due to the limited number of included studies, meta-analysis was not performed.

### Cumulative Meta-Analysis of the Association Between PD-L1 in TCs and Prognosis

**Figure 4:**
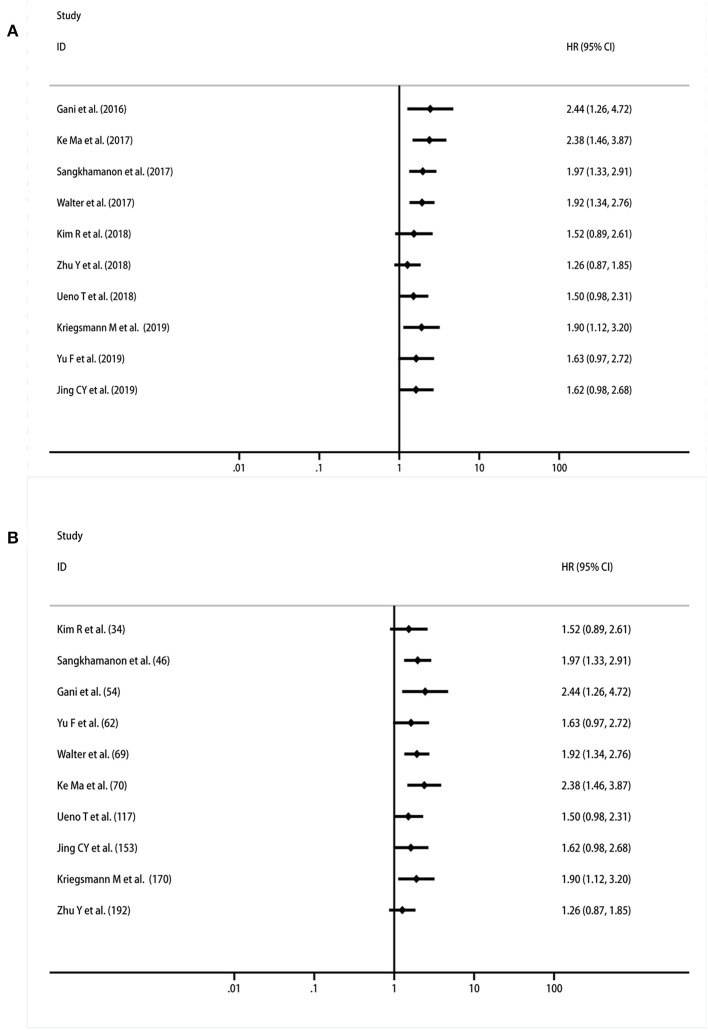
Cumulative meta-analysis of the association between PD-L1 expression in TCs and OS. **(A)** By years; **(B)** by sample size.

A cumulative meta-analysis was performed based on the publication year and sample size to investigate the trends in the results. The results indicated that the significant correlation between PD-L1 expression in TCs and OS became increasingly stable, starting with the study performed by Yu et al. (31) (Figure 4A). Regarding the trend associated with the sample size, the findings were still unstable when Zhu's research was reported, and the results became inconclusive (28) (Figure 4B).

### Subgroup Analysis of the Prognostic Effect of PD-L1 in TCs

**Figure 5:**
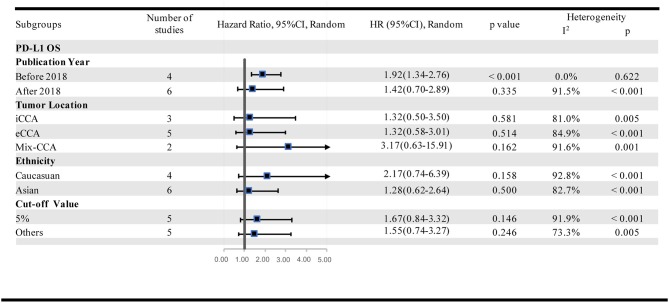
Subgroup analyses of the association between PD-L1 expression in TCs and OS.

Further subgroup analyses were performed to investigate the potential sources of heterogeneity among studies. This analysis was performed by stratifying the studies according to the publication year (before 2018 and after 2018), tumor location (iCCA and eCCA), patient ethnicity (Asian and Caucasian) and cut-off values (5% and others). Subgroup analysis regarding the tumor location showed that high-PD-L1 expression in TCs had no significant association with OS (OS for iCCA: HR = 1.32, 95% CI [0.50–3.50], *p* = 0.581; eCCA: HR = 1.32, 95% CI [0.58–3.01], *p* = 0.514; iCCA+ eCCA mixed: HR = 3.17, 95% CI [0.63–15.91], *p* = 0.162). Further, we conducted subgroup analyses based upon ethnicity. No significant association between PD-L1 and OS in Asian populations (HR = 1.28, 95% CI [0.62–2.64], *p* = 0.500) or in Caucasian populations (HR = 2.17, 95% CI [0.74–6.39], *p* = 0.158) was observed. To further restrict the prognostic effect with the same cut-off value, we performed subgroup analyses based on the method that was adopted to define the positivity of PD-L1 expression. However, the pooled outcomes were not significant in studies applying a 5% cut-off value (HR = 1.67, 95% CI [0.84–3.32], *p* = 0.146) and other cut-offs (HR = 1.55, 95% CI [1.55–3.27], *p* = 0.246). It was interesting that the results were inconsistent among the subgroups that were divided by the publication year (before 2018: HR = 1.92, 95% CI [1.34–2.75], *p* < 0.001; after 2018: HR = 1.42, 95% CI [0.70–2.89], *p* = 0.335). In addition, the between-study heterogeneity declined to some degree in some subgroups (Figure 5). There were too few studies that reported DFS stratified by PD-L1 in TCs and OS stratified by PD-L1 in ICs. For this reason, subgroup analysis was not possible or available for these cohorts.

### Meta-Regression Analysis

**Figure 6:**
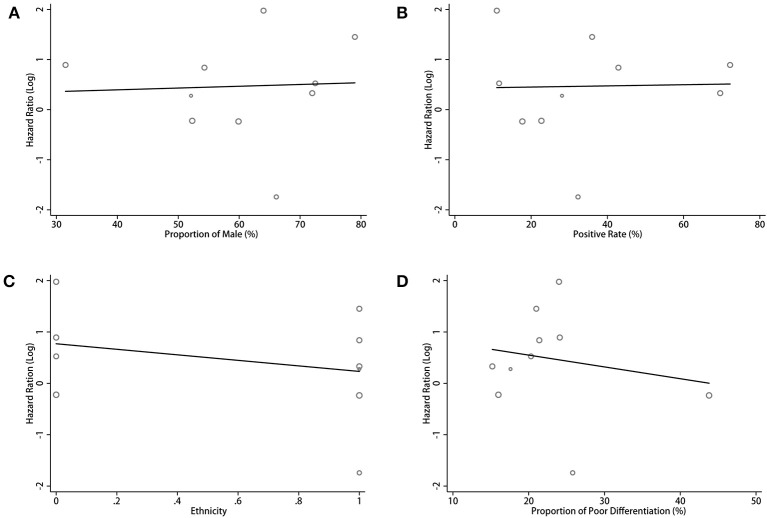
Meta-regression analysis between PD-L1 expression in TCs and OS in CCA. Bubble plot with a fitted meta-regression line of the log hazard ratio: **(A)** proportion of Male (%); **(B)** positive rate (%); **(C)** ethnicity; **(D)** proportion of poor differentiation (%).

For the pooled results of the association between PD-L1 expression in TCs and OS, the meta-regression analysis indicated a trend for the proportion of male, patient ethnicity, positive rate of PD-L1, and proportion of poor differentiation. However, no statistical significance was found (all *p* > 0.05, Figure 6; Table S1). Considering the significant heterogeneity in the pooled results, we estimated the contribution of different research characteristics to the level of heterogeneity (Table S1). However, there were no significant elements contributed to the level of heterogeneity. According to the results, the proportion of heterogeneity ranged from −8.83 to 19.00% (all *p* > 0.05). The remaining heterogeneity was high (τ^2^ range from 0.792 to 0.866). Because there was insufficient data in the included studies, other factors that may have caused the heterogeneity were not included in the meta-regression.

### Relationship Between PD-L1 and Clinicopathologic Characteristics

#### PD-L1 Expression in TCs

A total of 15 features from 10 studies that reported the correlation between PD-L1 expression in TCs and the clinicopathologic parameters of CCA were analyzed. The information for various clinicopathologic factors and their correlation with PD-L1 was demonstrated in Table 3. The results of the meta-analysis indicated that the expression levels of PD-L1 were significantly correlated with the AJCC TNM stage of disease (OR = 0.52, 95% CI [0.27, 0.99], *p* = 0.09). The other parameters did not reach statistical significance.

**Table 3:** The relationship between PD-L1 in TCs and the clinicopathological features.

**Parameters**	**Number of studies**	**Test for association**			**Test for heterogeneity**		
		**OR**	**95%CI**	***P***	**Chi^**2**^**	***I*^**2**^%**	***P***
Gender (Male vs. Female)	7	1.09	[0.72, 1.67]	0.68	2.07	0	0.91
Age (<60 years vs. ≥ 60 years)	3	1.20	[0.71, 2.03]	0.50	0.38	0	0.83
Tumor size (<3 cm vs. ≥3 cm)	2	0.47	[0.19, 1.20]	0.12	0.00	0	1.00
( ≤5 cm vs. >5 cm)	2	1.03	[0.61, 1.74]	0.91	0.17	0	0.68
Tumor number (Single vs. Multiple)	2	0.80	[0.09, 7.08]	0.84	9.95	89.9	0.00
Lymph node metastasis (Present vs. Absent)	10	1.26	[0.71, 2.23]	0.44	19.31	53.4	0.02
Perineural invasion (Present vs. Absent).	6	1.17	[0.66, 2.07]	0.59	3.0	0	0.70
Vascular invasion (Present vs. Absent)	6	1.39	[0.67, 2.86]	0.38	9.59	47.9	0.09
Resection margin (R1/R2 vs. R0)	4	0.60	[0.29, 1.26]	0.18	1.68	0	0.64
Tumor differentiation (Well, Moderately vs. Poor)	9	0.70	[0.39, 1.26]	0.23	16.38	51.1	0.04
TNM stage (I, II vs. III, IV)	8	0.52	[0.27, 0.99]	0.04	12.51	44.1	0.09

#### PD-L1 Expression in ICs

There were 8 parameters extracted from 4 studies that reported the relationship between PD-L1 and the clinicopathologic parameters of CCA that were analyzed. The information is summarized in Table 4. However, the data from the eight indicators revealed no statistical significance.

**Table 4:** The relationship between PD-L1 in ICs and the clinicopathological features.

**Parameters**	**Number of studies**	**Test for association**			**Test for heterogeneity**		
		**OR**	**95%CI**	***P***	**Chi^**2**^**	***I*^**2**^%**	***P***
Gender (Male vs. Female)	3	0.68	[0.19, 2.44]	0.55	8.94	77.6	0.01
Age (<60 years vs. ≥60 years)	2	1.21	[0.61, 5.04]	0.58	0.07	0	0.80
Lymph node metastasis (Present vs. Absent)	4	1.29	[0.50, 3.31]	0.60	7.81	61.1	0.05
Perineural invasion (Present vs. Absent).	2	1.87	[0.68, 5.12]	0.22	0.34	0	0.56
Vascular invasion (Present vs. Absent)	2	1.00	[0.32, 3.08]	1.00	0.15	0	0.70
Resection margin (R1/R2 vs. R0)	2	1.84	[0.67, 5.04]	0.24	0.16	0	0.69
Tumor differentiation (Well, Moderately vs. Poor)	4	0.85	[0.32, 2.29]	0.75	8.56	65.0	0.04
TNM stage (I, II vs. III, IV)	4	0.55	[0.16, 1.90]	0.34	4.84	38.0	0.18

### Correlation Between PD-L1 Expression in TCs and CD3^+^ TILs

Limited data showed the association between PD-L1 expression in TCs and CD3^+^ TILs in the included studies. By pooling the data of four cohort from three studies (24, 26, 32), we found no significant correlation between PD-L1 expression and CD3^+^ TILs (OR = 1.56, 95% CI [0.22, 10.92], *p* = 0.66) and high heterogeneity (*p* = 0.03; *I*^2^ = 73.0%).

### Sensitivity Analysis and Publication Bias

**Figure 7:**
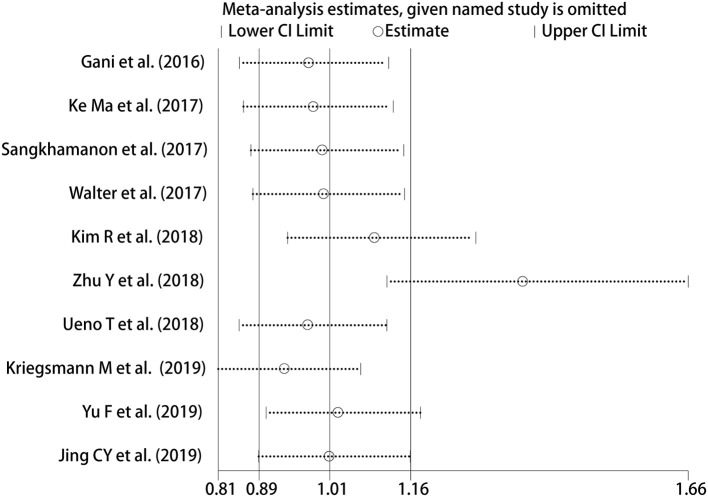
Sensitivity analysis between PD-L1 expression in TCs and OS.

**Figure 8:**
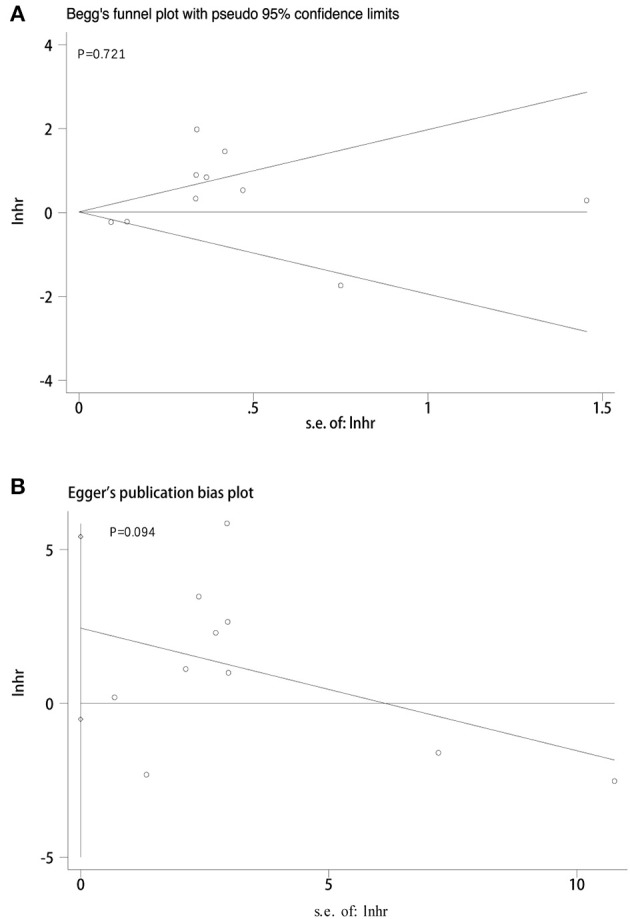
Publication bias tests between PD-L1 expression in TCs and OS. **(A)** Begg's funnel plot; **(B)** egger's funnel plot.

We adopted a random effects model in sensitivity analyses, deleting each study in each turn, to further determine the robustness of the prognostic role of PD-L1. As shown in Figure 7, the results of the pooled HRs remained changed when any study was deleted except for Zhu's study. When Zhu's study was deleted, high-PD-L1 expression in TCs had an unfavorable prognostic effect for OS in patients with CCA (HR = 1.36, 95% CI [1.11–1.66]). These results indicated that the association between PD-L1 expression in TCs and OS was not robustly significant. Because of the limited number of studies investigated the correlation between PD-L1 in TCs and DFS, the sensitivity analyses were not performed. The Begg's funnel plot and Egger's funnel plot were only performed to assess the publication bias of the correlation of PD-L1 expression in TCs and OS. As shown in Figure 8, the results of Begg's test and Egger's test showed no sign of publication bias for OS, with *p*-values of 0.721 and 0.094, respectively.

## Discussion

Cholangiocarcinoma, a highly aggressive tumor, is characterized by a paucity of effective therapy and a dismal prognosis. Recently, the introduction of immune checkpoint inhibitors including PD-1 and PD-L1 inhibitors has revolutionized the treatment landscape for many solid tumors. Recent clinical trials involving anti-PD-1 and anti-PD-L1 monoclonal antibodies (mAbs) have shown efficacy in various malignant tumors, with responses strongly correlated with PD-L1 expression, as assessed by immunochemistry (33–35). PD-L1 overexpression has been noticed in various solid tumors, and several studies have concluded that the expression of PD-L1 plays an important role in regulating the T-cell-mediated antitumor response and poor prognosis (36–39). However, the correlation of the expression of PD-L1 and the prognosis remains controversial for patients with CCA. Several studies have shown that positive PD-L1 expression is correlated with a significantly worse OS or DFS (22, 23, 28, 29), but other studies did not support this finding (24–27, 30–32). In the present meta-analysis, which was based on 11 studies with 1,066 patients, the results showed a trend that high PD-L1 expression indicated a poor OS, but the result was not statistically significant. For DFS, although the pooled result was not statistically significant, it trended toward being significant that high PD-L1 expression indicated improved DFS. In the evaluation of clinicopathological parameters, PD-L1 expression in TCs was significantly correlated with the AJCC TNM stage of CCA, suggesting that PD-L1 might be involved in the occurrence and progression of CCA. However, the other clinicopathologic characteristics did not reach statistical significance. Maybe that's because insufficient number of patients and the high level of heterogeneity render the analysis complicated. The relationship between PD-L1 and clinicopathologic characteristics needs further study in the future. To date, two regulatory mechanisms of PD-L1 induction have been proposed: intrinsic and extrinsic mechanisms (10, 40). Traditionally, it has been believed that the expression of PD-L1 can be driven by carcinogenic signaling pathways in cancer cells, which can lead to immune escape via innate immune resistance, which is associated with poor prognosis in patients (22, 23, 28, 29). Unlike the intrinsic mechanism, the extrinsic induction of PD-L1 at the tumor site is an adaptation to ongoing antitumor immunity, a process known as adaptive resistance (10). In this process, the infiltrating T cells can release interferon-γ (INF-γ). PD-L1 expression has been associated with the exposure to INF-γ which provides an efficient means for tumor cells to evade T cell immune surveillance (40). This process brings about a specific state of immune privilege which does not depend upon a systemic immune deficiency and is reversible with anti-PD-1 or anti-PD-L1 mAbs (41). From this point of view, the positivity of PD-L1 suggests the existence of immune surveillance and is linked with an improved prognosis (28, 31). The two mechanisms may coexist. Depending on the immunogenicity of cancer, the predominant mechanism may switch from one to the other at different times (42). The uncertainty of the prognostic role of PD-L1 tends to be an integrated effect of these two mechanisms.

Moreover, it is now increasingly accepted that, rather than working alone, cancer cells develop close interactions with the extracellular matrix, stromal cells, and immune cells that together form the TME. PD-L1 can be expressed on various cell types within TME, including tumor cells, epithelial cells, endothelial cells and ICs such as TILs, TAMs and other immune cells (34, 43). The expression of PD-L1 in ICs is also an indicator of a higher response rate to PD-L1/PD-1 checkpoint blockade therapy has been shown in some clinical trials (34, 44). It has also been proved that the expression of PD-L1 in immune cells was correlated with prognosis in several human cancers (45, 46). Although we pooled the results of PD-L1 expression in immune cells, our findings suggested that PD-L1 was not significantly associated with clinicopathologic characteristics. As only two studies' survival data was available, meta-analysis was not performed to evaluated the prognostic role of PD-L1 in ICs. Ma et al. reported that PD-L1 expression in ICs was associated with OS, but Walter et al. found that PD-L1 expression in ICs was not correlated with survival. Considering that tumor cells are not the only cells to express PD-L1, other types of cells within TME should be studied in the future. And the results of PD-L1 expression in other cells should be compared with tumor cells to determine which method has the best correlation with prognosis. If possible, the expression of PD-L1 in multiple types of cells should be detected to improve the accuracy of prognosis assessment.

Some studies have suggested that PD-L1-positive tumors had prominent immune cell infiltration in CCA, such as CD3^+^ TILs (represent overall T cells), CD8^+^ TILs (represent cytotoxic T cells), and tumor-associated macrophages (TAMs) (CD68^+^, CD163^+^) (28, 31, 32). This result has also been observed in other malignances (47–51), which may suggest the possibility of the adaptive immune resistance mechanism. However, the other two studies revealed that PD-L1 expression was not correlated to CD3^+^ TILs (24, 26). Then the pooled results showed no significant correlation between PD-L1 expression and CD3^+^ TILs. As aforementioned, unlike the adaptive immune resistance mechanism, the expression of PD-L1 can be driven by carcinogenic signaling pathways in cancer cells in the innate immune resistance mechanism (10). We assumed that PD-L1 was not correlated to CD3^+^ TILs can be explained through this mechanism. In a retrospective cohort of 435 biliary duct cancer patients, the investigators evaluated the TILs by IHC. The results showed that most commonly detected immune cells at the tumor site were CD8^+^ TILs, followed by CD4^+^ TILs; Foxp3^+^ regulatory T (Treg) cell were 12%. But the B cells and NK cells were rarely found. The correlation between Treg cell and PD-L1 were investigated in some studies (52, 53). The Treg cells, a subtype of T cells, can restrain the activity and proliferation of cytotoxic T cells (54). As a kind of immunosuppressive cell type within the TME, the Tregs expression was correlated with poor prognosis in some tumors (55–57). However, a study revealed that the CCA patients with tumor-infiltrating CD4^+^ T cells, CD8^+^ T cells, and Treg cells showed a significantly longer overall survival (58). Considering the complexity of the tumor microenvironment, and tumor heterogeneity, it is possible that PD-L1 expression should be combined with another marker such as CD8^+^ T cells infiltration, Treg cells or TAMs expression in order to gain more insights into their prognostic values in CCA patients. In addition to assess prognosis, the PD-L1 expression in tumor is also a significant biomarker to predict treatment reaction of PD-1/PD-L1 checkpoint blockade. Currently, some studies reported that PD-L1 positive colorectal cancers with high-level microsatellite instability (MSI-H) were sensitive to PD-1/PD-L1 checkpoint blockade therapy (59, 60). However, the findings of CCA were contrary (61). The result indicated that MSI status can serve as a predictive biomarker for PD-1/PD-L1 checkpoint blockade therapy in CCA, but the incidence of MSI-H in CCA is low (1.3%, 4/308) according to the results of other study (24). Therefore, PD-L1 expression combined with other biomarkers may assist clinicians to stratify CCA patients for anti-PD-1/PD-L1 therapy.

Considering that significant heterogeneity was observed in the association between PD-L1 in TCs and OS, subgroup analysis and meta-regression analysis was performed. High PD-L1 predicted a worse prognosis in the studies published before 2018, but no significant correlation was found between PD-L1 and OS in the studies that were published after 2018. As the result of a cumulative meta-analysis that was performed based on the publication year, the results reverted to having no statistical significance and became increasingly stable after Kim's studies were reported in 2018. We noted that the sample size of the articles before 2018 was generally smaller. Additionally, the positivity rates of PD-L1 expression in TCs ranged from 11.6 to 72.2%, which are higher than those in the studies that were published after 2018. We believe that the earlier studies might have employed an invalid PD-L1 antibody, while the antibodies employed in more recent studies can distinguish between cytoplasmic and membranous patterns of PD-L1 staining (24, 27–31). The cut-off values used to evaluate the positivity of PD-L1 expression were varied in each study before 2018. The reasons listed above might have confounded the heterogeneity among the studies and the different prognostic role of PD-L1 in CCA. However, other factors increased the heterogeneity as well. The expression patterns of PD-L1 were different in each study. Kim's study included PD-L1 staining in the cell cytoplasm (27), while Ueno's study included PD-L1 staining in the cell membrane and cytoplasm as well (29). However, the other studies included PD-L1 staining in the cell membrane. If the expression pattern of PD-L1 in CCA cannot be distinguished, the positivity rate of PD-L1 may be inaccurate. Additionally, we tried to pool the results from patients with similar genetic background (Asian or Caucasian) to reduce the heterogeneity. To our disappointment, the between-study heterogeneity was still high. In addition, the various methods of IHC staining and the inconsistent follow-up periods was likely to be another contributing factor. Nevertheless, our meta-regression analysis did not determine a factor that significantly contributed to the level of heterogeneity, and the roles of the aforementioned factors deserve further investigation. To normalize the methods listed above, a worldwide standard for the detection of PD-L1 expression should be established. Additionally, some important baseline patient characteristics, such as CA19-9, CEA, and microvascular invasion, must be taken into account when analyzing the prognostic role of PD-L1.

The sensitivity analyses implied that the correlation between PD-L1 expression in TCs and OS was not robustly stable. The results of the pooled HRs changed when Zhu's study was omitted (28). The results of Zhu's study indicated that PD-L1 was a positive prognostic factor for CCA patients, which is similar to Yu's study. The investigators determined that PD-L1 was correlated with CD8^+^ T-cell infiltration through measuring the mRNA levels of PD-L1 and CD8A via quantitative PCR. Additionally, INF-γ mRNA was also correlated with PD-L1 expression (28). The positive correlation among PD-L1, CD8^+^ T-cell and INF-γ expression provided a direct evidence of the adaptive resistance mechanism. In addition, the sample size of each study that we included was relatively small. Among the 11 studies, only four studies had more than 100 patients (24, 28–30). The sample size of Zhu's study was 192, which was the largest one among the included studies. According to the results of the cumulative meta-analysis performed based on the sample size, the results became unstable and remained inconclusive starting when Zhu's study was reported. In the future, more prospective studies with larger sample sizes should be developed and carried out.

The recurrence rates of CCA patients after surgery remain high (49–64%), and recurrences usually occur within 2–3 years post resection (62). However, only three studies investigated the correlation between PD-L1 expression and DFS (27, 28, 31). In addition, four studies focused on the association of PD-L1 expression in ICs and OS (22–24, 26), but the data of only two studies could be used (23, 26). Therefore, future research should focus on the significance not only between PD-L1 and OS but also between PD-L1 and DFS in CCA patients. In addition, we highly recommend that further studies be performed to investigate the prognostic roles of PD-L1 expression in other cells in addition to in tumor cells. Although the pooled results of the correlation between PD-L1 expression in TCs and OS was not robustly stable, combined with the association between the DFS and PD-L1 in TCs and the association of PD-L1 in ICs with OS, we believe that our study reveals profound statistical evidence about significant prognostic role of PD-L1 in CCA.

To our knowledge, the present study is the first meta-analysis to evaluate the prognostic value of positive-PD-L1 expression in CCA patients. Additional information regarding the interstudy heterogeneity issues was obtained by subgroup analysis and meta-regression analysis. However, several limitations still existed in our study. First, all the included studies were retrospective and data from prospective studies were lacking. Large multi-center prospective cohorts are needed to investigate the predictive roles of PD-L1 expression in CCA. Second, heterogeneity existed in our meta-analysis, which we believe was the result of many factors. However, the factors that significantly contributed to heterogeneity were not identified by the meta-regression analysis. Third, the number of included studies reporting PD-L1 expression in ICs and the association between PD-L1 expression in TCs and DFS were relatively small. Because there was insufficient number of eligible studies, we did not conduct subgroup analysis, sensitivity analysis, and publication bias analysis. Finally, as only one paper reported the role of sPD-L1 in serum in CCA patients, PD-L1 expression was detected by IHC in tumor tissues in the included studies in our meta-analysis (63). More researchers have confirmed the prognostic significance of sPD-L1 in solid tumors (64–66). We suggest that further studies be performed to investigate the prognostic roles of sPD-L1 in CCA patients.

## Conclusion

Despite these limitations, our meta-analyses revealed that PD-L1 expressed in TCs was significantly correlated with the AJCC TNM stage of CCA. Based on the included studies, we found that PD-L1 indeed expressed in both TCs and ICs in CCA patients, raising the possibility of the use of anti-PD-1/PD-L1 therapy for CCA patients. In contrast, expression of PD-L1 did not seem to be associated with patient outcome in our study. The prognostic role of PD-L1 in CCA demands further investigation.

## Data Availability

Publicly available datasets were analyzed in this study. This data can be found here: PubMed, Embase, the Cochrane Library, Web of Science, and Google Scholar.

## Author Contributions

GX designed the study, performed the literature search and screening, performed the data analyses, and drafted the manuscript. LS and YL designed the study, extracted the literature and data, analyzed the extracted data, and took part in the writing of manuscript. FX, HY, XZ, and SD assisted in the designing of the study, performed the literature search and screening, assisted in the data analyses, and took part in the writing of manuscript. YM and HX designed the study and supervised the study. All authors approved the final version of this study.

### Conflict of Interest Statement

The authors declare that the research was conducted in the absence of any commercial or financial relationships that could be construed as a potential conflict of interest.
